# Evaluation of In Vitro and In Silico Anti-Alzheimer Potential of Nonpolar Extracts and Essential Oil from *Mentha piperita*

**DOI:** 10.3390/foods12010190

**Published:** 2023-01-01

**Authors:** Manel Srief, Moustafa Bani, El Hassen Mokrani, Imad Mennai, Mehdi Hamdi, Abdenour Boumechhour, Mohamed Abou Mustapha, Mouna Derdour, Messouad Kerkatou, Mohamed El-Shazly, Chawki Bensouici, Gema Nieto, Salah Akkal

**Affiliations:** 1 Biotechnology Laboratory, National Higher School of Biotechnology, Constantine 25000, Algeria; 2 Biotechnology Research Center, Ali Mendjli New City UV 03, BP E73, Constantine 25016, Algeria; 3 Laboratory of Applied Biochemistry, Department of Biochemistry and Cellular and Molecular Biology, Faculty of Natural and Life Sciences, University Mentouri Brothers Constantine 1, Constantine 25017, Algeria; 4 Research Unit Valorisation of Natural Resources, Bioactive Molecules and Physicochemical and Biological Analysis, University Mentouri Brothers Constantine 1, Route d’Ain El Bey, Constantine 25000, Algeria; 5 Centre for Scientific and Technical Research in AnalysisPhysico-Chemicals (C.R.A.P.C), BP384, Bou-Ismail, Tipaza, Algiers 42004, Algeria; 6 Department of Pharmacognosy, Faculty of Pharmacy, Ain-Shams University, Cairo 11566, Egypt; 7 Pharmaceutical Biology Department, Faculty of Pharmacy and Biotechnology, The German University in Cairo, Cairo 11432, Egypt; 8 Department of Food Technology, Food Science and Nutrition, Faculty of Veterinary Sciences, Regional Campus of International Excellence “Campus Mare Nostrum”, University of Murcia, 30071 Murcia, Spain; 9 Unit of Recherche Valorisation of Natural Resources, Bioactive Molecules and Analyses Physicochemical and Biological (VARENBIOMOL), Faculty of Science, Department of Chemistry, University Mentouri-Constantine 1, Constantine 25000, Algeria

**Keywords:** *Mentha piperita*, antioxidant activities, anticholinesterase, molecular docking

## Abstract

The anticholinesterase and antioxidant activities with chemical composition and molecular docking of essential oil and nonpolar extracts of *Mentha piperita* were evaluated using enzymatic and chemical methods. Molecular docking tools were used to explain the interaction of the major chemical constituents with the enzymes. GC/MS analyses revealed that the main compounds in *M. piperita* essential oil were l-menthone (43.601%) followed by pulegone (21.610%), linolenic acid (25.628%), and l-menthone (10.957%), representing the major compounds of the petroleum ether extract. Imidazoquinoline (7.767%) and 17-N-acetyl-oroidine (5.363%) were the major constituents of the chloroform extract. Linolenic acid (19.397%) and l-menthone (6.336%) were the most abundant compounds in the hexane extract. The *M. piperita* essential oil and nonpolar extracts showed moderate antioxidant activity. The essential oil showed the most promising anticholinesterase activity with IC_50_ = 10.66 ± 0.12 µg/mL and IC_50_ = 16.33 ± 0.03 µg/mL against acetylcholinesterase (AChE) and butyrylcholinesterase (BChE), respectively, close to galantamine in AChE and more active in BChE, followed by the interesting activity in the petroleum ether extract with IC_50_ = 23.42 ± 3.06 µg/mL in AChE and IC_50_ = 62.00 ± 3.22 µg/mL in BChE. The docking experiments showed that among the seven major identified compounds, N-acetyl-17-oroidine showed the highest binding score (63.01 in AChE and 63.68 in BChE). This compound was found to bind the catalytic and peripheral sites, resulting in more potent inhibitory activity than galantamine, which only binds to the catalytic site. These findings suggested the possible use of *M. piperita* essential oil and nonpolar extracts as a potential source of alternative natural anti-Alzheimer compounds.

## 1. Introduction 

Alzheimer’s disease (AD) is a progressive neurological disorder leading to impairment in memory, language skills, judgment, and orientation. Recent results indicated that acetylcholinesterase (AChE) neurotransmission suppresses the formation of β-amyloid, one of the hallmarks of AD [1,2]. Oxidative stress can also promote AD in addition to a group of other serious diseases such as arthritis, atherosclerosis, cardiovascular disorders, and cancer [3]. Cerebral tissues have a low amount of antioxidants and a high amount of polyunsaturated fatty acids in the membranes of their neurons, and they need a lot of oxygen to function. Thus, cerebral tissues are highly affected by oxidative stress from free radicals [4]. The inhibition of acetylcholinesterase (AChE) and butyrylcholinesterase (BChE) is one of the most effective therapeutic approaches to treat the symptoms of AD [5]. Currently, only four therapeutic agents were approved to tackle AD [1]. Most of these drugs show serious side effects including hepatoxicity, bradycardia, and gastrointestinal disturbance [6]. Synthetic antioxidants such as butylated hydroxyanisole (BHA) and butylated hydroxytoluene (BHT) were previously used to overcome high oxidative stress, but they showed some side effects and can be potentially toxic [7]. Natural antioxidants emerged as valuable alternatives to synthetic derivatives with potent activity associated to fewer side effects such as both acute and chronic health effects and damage to the nervous system and kidneys, and cancer risk.

It is commonly known that humanity has relied on certain medicinal plants to fulfill its therapeutic demands. The World Health Organization stated that over 60% of developing countries’ populations use medicinal plants to treat many disorders such as cancer, diabetes mellitus, and cardiovascular and neurodegenerative diseases [8]. With the current changes in the lifestyle of developing countries, a group of serious diseases emerged due to excessive oxidative stress including neurodegenerative disorders, cancer, and metabolic and cardiovascular diseases. Many enzymes including AChE and BChE participate in the development and progression of these diseases [9]. Therefore, it is critical to seek out new and safe antioxidants, as well as plant-derived AChE and BChE inhibitors with better outcomes.

There are hundreds of subspecies and cultivars in the Lamiaceae family genus Mentha, which has roughly 42 species and 15 hybrids [10]. The plant is found in temperate regions of Eurasia, Australia, and Africa. Among the most common species is peppermint, *M. piperita*, which is characterized by its volatile oils used in the flavor, fragrance, and pharmaceutical industries [11]. *M. piperita* leaves contain up to 2% essential oil. Its oil is considered the second most important essential oil after citrus oil with about 14,000 tons produced annually and USD 300 million of revenue with menthol as the primary component. The oil and its components are used in pharmaceutical and cosmetical industries [12,13,14]. The principal volatile compounds of *M. piperita* are composed especially of menthol, menthone, menthofuran, carvone, menthyl acetate, D-limonene, and 1,8-cineole [15,16]. Other bioactive ingredients include bitter compounds and phenolic acids, especially caffeic acid, p-cumaric acid, ferulic acid, rosmarinic acid, caftaric acid, chlorogenic acid, m-coumaric acid, and o-coumaric acid, as well as flavonoids such as naringin, luteolin, and riboflavin, monoterpene epoxide such as cis-carvone oxide, tannins, and bitter principles [10,13]. The literature data revealed that this plant has an in vitro scale (cells and enzymes assays) antimicrobial, antimycotic, antioxidant, and anticancer effects, and at the in vivo scale (animal studies using laboratory-bred mice or rats), it showed a spasmolytic, analgesic, antiviral, anti-human immunodeficiency, anti-inflammatory, hepatoprotective, chemopreventive, anti-obesity, anti-diabetic, cardioprotective, and antiflatulence effects [11,12,13,14]. 

The aim of this research is to investigate the phytochemical constituents of essential oil and nonpolar extracts of *M. piperita* grown in northeast Algeria, using gas chromatography–mass spectrometry (GC-MS), and to explore the in vitro antioxidant properties using various methods. Furthermore, the anticholinesterase activity was evaluated using a combination of two complementary method systems: AChE and BChE. Molecular docking studies were also carried out to investigate the binding affinity of major compounds to the active sites of AChE and BChE.

## 2. Materials and Methods

### 2.1. Reagents and Chemicals Used 

The measurements of the activities were carried out using a 96-well microplate reader (PerkinElmer Multimode Plate Reader EnSpire) at the National Center for Biotechnology Research. The chemical products and reagents used were: Folin–Ciocalteu reagent (FCR), 1,1-diphenyl-2-picrylhydrazyl (DPPH), butylated hydroxylanisole (BHA), butylated hydroxyltoluene (BHT), quercetin, α-Tocopherol, ascorbic acid, neocuproine, 2,2′-azino-bis(3-ethylbenzothiazoline-6-sulfonicacid)-diammoniumsalt(ABTS), trichloroacetic acid (TCA), potassium ferricyanide (C_6_N_6_FeK_3_), phenanthroline, silver nitrate (AgNO3), trisodium citrate (Na_3_C_6_H_5_O_7_), acetylcholinesterase from electric eel (AChE, Type-VI-S, EC 3.1.1.7, 827.84 U/mg, Sigma), butyrlcholinesterase from horse serum (BChE, EC 3.1.1.8, 7.8 U/mg, Sigma), acetylthiocholine iodide, S-Butyrylthiocholine iodide, 5,5′-Dithiobis(2-nitrobenzoic) acid (DTNB), and galantamine, which were obtained from Sigma-Aldrich; and sodium carbonate, aluminum nitrate (Al(NO_3_)_3_, 9H_2_O), iron (III) chloride (FeCl_3_), sodium bicarbonate (NaHCO_3_), copper (II) chloride (CuCl_2_), potassium persulfate (K_2_S_2_O_8_), and potassium acetate (CH_3_CO_2_K), which were obtained from Biochem Chemopharma. All other chemicals and solvents were of analytical grade. 

### 2.2. Raw Material Extraction

The aerial parts of *M. piperita* were collected in 2020 from the Mila region (northeast Algeria). The plant was authenticated by Mr. Mohamed Kaabeche, a botanist at Ferhat Abbas University, Setif. A sample specimen was deposited in the herbarium (MEP 55/04/20) (Tables S1–S3). According to the *British Pharmacopoeia*, the essential oil was hydro-distilled for 3 h with a Clevenger apparatus before being stored at 4 °C until use. The aerial parts (60 g) of the plant were divided into three parts in equal weight (20 g) and separately extracted by maceration with 200 mL of solvents (chloroform, hexane, and petroleum ether). The extraction was performed three times with renewal of the solvent. The contents were then filtered through Whatman filter paper no.1. The filtrate was evaporated at 40 °C by a rotary evaporator and kept in a refrigerator at 4 °C until use.

### 2.3. GC-MS Analysis

Agilent GC-MS using an HP-5MS column was employed with a temperature programmed at 60 °C in isothermal conditions, and helium gas was used at a 0.5 mL/min rate flow. The MS parameters were: electron energy, 70 eV; ionization, 2 A; temperature, 280 °C; resolution, 1000 scan time, 5 s. The following temperature program was applied: 40 °C to 150 °C, for 8 min and 5 min, respectively, and at a rate of 5 °C/min to 260 °C for 15 min. Compounds were identified by comparing the mass spectra with the spectral libraries NIST and Wiley.

### 2.4. Colorimetric Total Phenolic and Flavonoid Content 

#### 2.4.1. Colorimetric Total Phenolic (CTP)

The total phenolic content was determined by the Folin–Ciocalteu method with slight modification [15] and the results were expressed in µg of gallic acid per mg of extract. 

#### 2.4.2. Colorimetric Total Flavonoid (CTF)

The total flavonoids were determined using the aluminum nitrate method [16] and the results were expressed in µg of quercetin per mg of extract. 

### 2.5. Colorimetric Antioxidant Capacity

In plants, antioxidants can be found in many different groups and forms, including carotenoids, phenolic compounds, benzoic acid derivatives, flavonoids, and coumarins. For the measurement of antioxidant content and total antioxidant capacity, various spectrophotometric techniques are used to provide a thorough profile of the antioxidant content and capacity of the tested samples. In this study, the antioxidant capacity of the essential oil and the nonpolar extracts was assessed using six different methods including DPPH^•^, reducing power, phenanthroline, silver nanoparticle, ABTS^•+^, and CUPRAC, compared with five standards: BHA, BHT, ascorbic acid, quercetin, and α-tocopherol. 

#### 2.5.1. Colorimetric DPPH Assay

The DPPH assay was evaluated according to the method of [17] and BHA, ascorbic acid, quercetin, α-tocopherol, and BHT were used as positive standards for comparison of the obtained results.

#### 2.5.2. Colorimetric Reducing Power Assay

The antioxidant capacity of the essential oils and the nonpolar extracts was evaluated using the potassium ferricyanide method [18] and BHA, ascorbic acid, quercetin, α-tocopherol, and BHT were used as positive standards for comparison of the obtained results.

#### 2.5.3. Colorimetric Phenanthroline Assay

The activity was determined by the phenanthroline method of [19] and the results were given as A_0.50_ (µg/mL). BHA, ascorbic acid, quercetin, α-tocopherol, and BHT were used as positive standards for the comparison of the obtained results.

#### 2.5.4. Colorimetric Silver Nanoparticle Assay 

The silver nanoparticle activity was evaluated according to the method described by [20] and the results were given as A_0.50_ (µg/mL). BHA, ascorbic acid, quercetin, α-tocopherol, and BHT were used as positive standards for the comparison of the obtained results.

#### 2.5.5. Colorimetric ABTS Assay 

The ABTS^•+^ activity was evaluated by the method in [21] and the results were given as IC_50_. BHA, ascorbic acid, quercetin, α-tocopherol, and BHT were used as positive standards for the comparison of the obtained results.

#### 2.5.6. Colorimetric Cupric-Reducing Antioxidant Capacity (CUPRAC) 

The cupric-reducing antioxidant capacity was measured by the method of [22] and the results were given as A_0.50_ (µg/mL). BHA, ascorbic acid, quercetin, α-tocopherol, and BHT were used as positive standards for the comparison of the obtained results.

### 2.6. Colorimetric Cholinesterase Inhibition Activity

The Ellman method was used to estimate the inhibitory potential of the studied samples [23] and galantamine was used as a positive standard for the comparison of the obtained results.

### 2.7. Statistical Analysis

All estimated parameters were subjected to one-way analyses of variance (ANOVA), with the various tested extracts and essential oils serving as fixed factors. This was conducted in triplicate. An analysis of the differences in means between each treatment was carried out using the Tukey’s multiple range test whenever the ANOVA test was statistically significant (*p* < 0.05).

### 2.8. Molecular Docking

A molecular docking study was conducted using GOLD version 5.2.2 [24]. Before running the docking experiments, potential ligands were built and prepared with the Maestro version 11.3 of Schrodinger’s LigPrep module [25]. For each ligand, several structures (up to 32) with various tautomers, protonation states at pH = 7.4 ± 1, and enantiomers were generated. All of these conformations were minimized and compiled as mol2 files. The crystal structures of AChE (4M0E) and BChE (2XQF) were downloaded from the Protein Data Bank (http://www.rcsb.org/ accessed on 20 April 2022) [26,27]. For each enzyme, the residues within a radius of 6 Å around the co-crystal ligand were considered active sites. This selection was refined by adding every residue beyond 6 Å considered important for the continuity of the cavity [28]. Then, all hydrogen atoms were added. Thereafter, the protonated states were defined and the side-chain orientation of the active site’s residues was controlled using Schrödinger’s protein preparation wizard. Finally, the intramolecular energy was lowered, and a mol2 file was saved. Enzyme ligand interactions were visualized using Maestro software version 11.1 [29]. 

The co-crystallized ligand of each enzyme was removed and redocked in the active site using GOLD in which the target atoms are fixed, and the ligands are flexible. After successful redocking of the co-crystallized ligand (RMSD value < 1 Å), the same docking parameters were used for *M. piperita* major compounds [30]. 

## 3. Results 

### 3.1. GC/MS of Essential Oil and the Nonpolar Extracts 

Various components present in *M. piperita* were detected by the GC-MS and are shown in the Supplementary Materials (Tables S1–S4). A total of 113 compounds in essential oil, 118 constituents in the petroleum ether, 41 constituents in chloroform extract, and 85 compounds in hexane extract were detected. The identification of compounds was based on their retention times in comparison with matching peaks available in the NIST and Wiley mass spectral libraries, as well as by comparing the fragmentation pattern of the mass spectra and their retention indices with those reported in the literature by the NIST Standard Reference Database (version 2.4).

### 3.2. Total Phenolic and Flavonoid Content Determination 

The total phenolic content was expressed as micrograms of gallic acid equivalents per milligrams of extract (μg GAE/mg) and the total flavonoid content as micrograms quercetin equivalents per milligram of extract (μg QE/mg). The results are summarized in Table 1.

### 3.3. Antioxidant Activities

A total of six in vitro antioxidant tests (DPPH^•^, reducing power, phenanthroline, silver nanoparticle, ABTS^•+^, and CUPRAC assays) were used and compared with five standards: BHA, BHT, ascorbic acid, quercetin, and α-tocopherol. The results were obtained using a linear regression analysis, and the IC_50_ and A_0.5_ values are listed in Table 1 as the mean values ± SD of three measurements. In almost all tests, the IC_50_ and A_0.50_ values were far from the standard ones, except for the CUPRAC properties and the ABTS radical cation-scavenging activity that showed moderate effects.

### 3.4. Enzyme Inhibitory Activities

**Figure 1:**
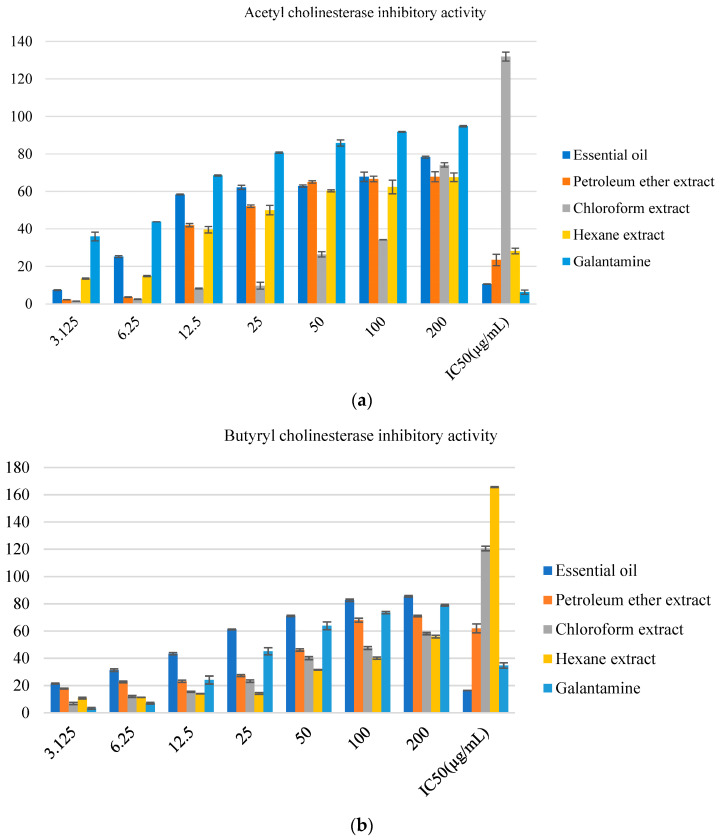
(**a**) Cholinesterase inhibitory of nonpolar extracts and essential oil of *Mentha piperita.* (**b**) Cholinesterase inhibitory of nonpolar extracts and essential oil of *Mentha piperita*.

In addition to the antioxidant activity, the cholinesterase enzyme inhibitory effects were evaluated with galantamine as the positive control and the inhibition enzyme was expressed as IC_50_ (μg/mL) and is presented in Figure 1a,b. The studied samples revealed an inhibitory activity of AChE and BChE in a dose-dependent manner and the essential oil showed the most potent inhibitory.

### 3.5. Molecular Modeling

**Figure 2:**
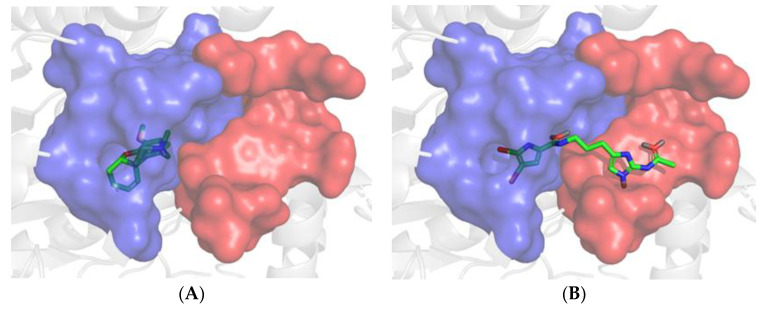
Positioning of galantamine (**A**) and N-acetyl-17-oroidine (**B**) in AChE-active site. Each compound is presented in its most plausible position after protein–ligand docking via GOLD. For the sake of clarity, residue Tyr341, which covers the ligand in the pocket, is left out. The cavity’s CAS and PAS regions are depicted in blue and red, respectively. The color codes of the ligand are green for carbon, red for oxygen, and blue for nitrogen atoms. PyMOL was used to draw images.

**Figure 3:**
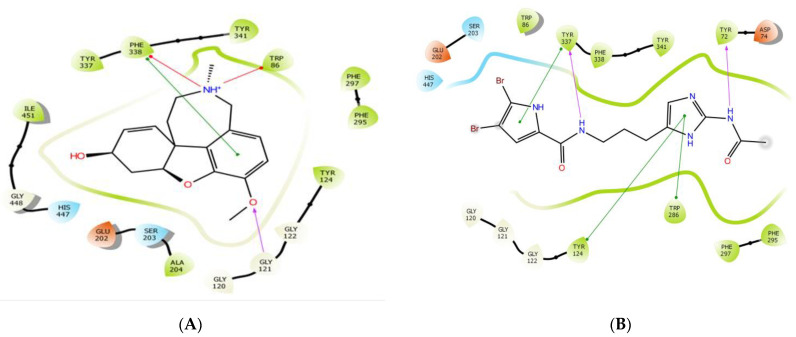
Prediction binding mode of galantamine (**A**) and N-acetyl-17-oroidine (**B**) into the whole AChE active pocket. Red lines indicate π–cation interactions, green lines π–π stacking, and purple arrows show the direction of hydrogen bonding from the donor to the acceptor. The diagrams were made with the Ligand Interaction Diagram script from the Schrödinger Suite.

**Figure 4:**
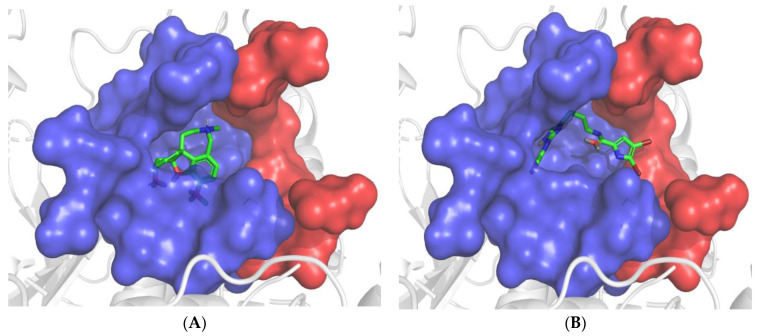
Prediction binding mode of galantamine (**A**) and N-acetyl-17-oroidine (**B**) into the whole BChE active pocket. The same color pattern used in Figure 3 was used.

**Figure 5:**
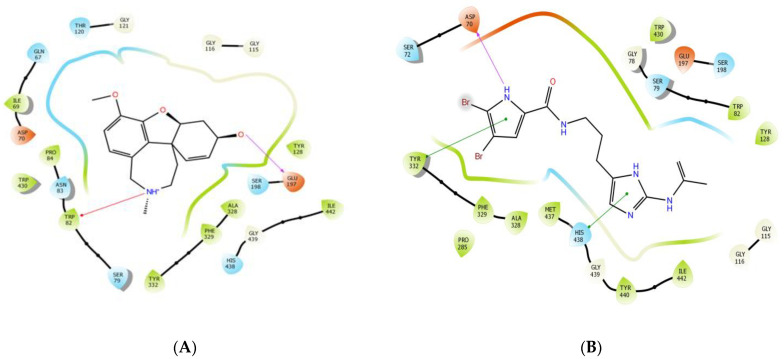
Prediction binding mode of galantamine (**A**) and N-acetyl-17-oroidine (**B**) into the entire BChE-active pocket. The same color code used in Figure 3 was applied.

The affinity of the seven major compounds with the studied enzymes (AChE and BChE) were evaluated, and the docking scores are listed in Table 2. N-acetyl-17-oroidine exhibited the higher enzymatic inhibition. For this, its binding mode and amino acid interactions were studied with AChE (presented in Figure 2 and Figure 3) and with BChE in Figure 4 and Figure 5. The results are summarized in Table 3. 

## 4. Discussion 

This is a preliminary study of the chemical composition, antioxidant and anticholinesterase effects, and in silico modeling of the essential oil and nonpolar extracts from Algerian *M. piperita*. The results of this study show that the essential oil was characterized by l-menthone, pulegone, and 1,8-cineole; this result is in good agreement with previous studies, with a slightly different profile [31,32], mainly the lower amount of germacrene D, (E)-caryophyllene, limonene, and p-cymene. These slight variations of the essential oil composition might be due to genotype–environment interactions as well as the phonological stage of the studied species, which causes plants to produce some chemical biomolecules as an adaptive response to environmental changes [33]. For the nonpolar extracts, the results showed the presence of the following major constituents: linolenic acid, l-menthone, and pulegone in petroleum ether extract; imidazoquinoline, 17-N-acetyl-oroidine, and l-menthone in the chloroform extract; and linolenic acid, l-menthone, and neicosane in the hexane extract. The present results are in good agreement with previous studies that recorded that the main constituent of the most widely used Mentha species are menthone, linolenic acid, menthol, limonene, isomenthone, menthyl acetate, carvone, β-pinene, 1,8-cineole, pulegone, and piperitone oxide [13,34]. 

The researchers reported that the total phenolic content of the hexane, chloroform, and petroleum ether extracts (11.48 ± 0.39, 18.53 ± 0.34, and 155.79 ± 0.53 µg GAE/mL, respectively) were found to be relatively low in the present study [35]. The obtained results of the flavonoid contents were lower than those reported by other authors in hexane, chloroform, and ethanol extracts (209.08 ± 0.83, 122.33 ± 0.39, and 300.06 ± 1.65 µg CE/mL, respectively) [36].

According to the literature data, extracts of ethyl acetate, methanol, water, and ACN solvents had the highest content of total phenols and flavonoids [12]. This could be explained by the fact that polar solvents have a higher affinity with these components than nonpolar solvents, which has already been confirmed in a previous study on Mentha species [36]. The quantitative differences in the phenolic content might be attributed to the differences in the extraction methods, the used standard solution, geographical location and climate conditions [35]. Antioxidants follow two different mechanisms: hydrogen donation (hydrogen atom transfer, HAT) and electron transfer (single electron transfer, SET). In our study, we used several methods (DPPH^•^, ABTS^•+^, CUPRAC, reducing power, phenanthroline, and silver nanoparticle (SNP)). Knowing that the difference between these assays is in the mode of action and the reaction mechanism, our aim was to obtain more information about the antioxidant activity of the studied extracts.

In the DPPH assay, all samples showed feeble activity (IC_50_ > 800 µg/mL) distant to the standards. These results were in agreement with previous results that showed an IC_50_ of 860 µg/mL in nonpolar extracts [37]. Similarly, other works showed that the highest activity was obtained in the aqueous extract, while the lowest activity was exhibited by the chloroform and hexane extracts [34,38].

Regarding the reducing power assay, *M. piperita* essential oil and nonpolar extracts showed a weak ability to reduce iron with A_0.50_ > 200μg/mL in comparison with the results of the α-tocopherol, BHA, BHT, ascorbic acid, and quercetin used as standards, which showed a strong reducing effect of iron. These results are in agreement with those reported in the literature [34], indicating that the hexane and DCM extracts exhibit the weakest reducing power. The low activity in both DPPH radical scavenging and reducing power assays were strongly associated with the phenolic content of the extracts. In many studies, a high positive correlation between anti-DPPH activity, reducing power, and total phenolic content was demonstrated [34,39].

As in the previous antioxidative assays, the phenanthroline test showed that the essential oil, hexane, and petroleum ether (A_0.50_ > 200 μg/mL) had low activity. However, the chloroform extract (A_0.50_ = 140.11 ± 0.98 μg/mL) demonstrated the most potent antioxidant effect, but remained weak when compared with the standards. The same result was obtained in the silver nanoparticle assay: the highest antioxidant activity was found with the chloroform extract (A_0.50_ = 124.97 ± 0.41 μg/mL). It was more potent than the BHT and ascorbic acid used as the standards (A_0.50_ > 200 μg/mL). In terms of similarity in the mode of action of the silver nanoparticle and phenanthroline assays, our results are in total agreement with the work carried out by [40], which showed a moderate activity in iron (Fe^3+^) reduction (˂0.5 mmol ascorbic acid/g sample) for the petroleum (PE) and dichloromethane (CH_2_Cl_2_) extracts, respectively.

In the ABTS assay, all of the samples exhibited low activity compared with the five positive standards. These results were slightly lower when compared with other studies that state that the percentage of ABTS-scavenging capacity of the essential oil of *M. piperita* was found to be 80.6 ± 1.45% diluted 20-fold in methanol [37]. Despite the low content of phenolic compounds, no correlation was found between these and the ABTS^•+^ activity [41]. The ABTS^•+^ activity was attributed to certain monoterpene alcohols, ethers, ketones, and aldehydes and some nonpolar bioactive compounds such as the pigments [37,42,43,44].

Regarding CUPRAC properties, the chloroform extract (A_0.50_ = 89.61 ± 0.9 μg/mL) showed the most potent activity close to α-tocopherol. The order of activity was essential oil < hexane < petroleum ether < chloroform. The CUPRAC assay results were consistent with those previously reported [34], finding that the nonpolar extracts such as hexane and dichloromethane presented the weakest reducing power [34]. The samples showed a little more activity in both methods (CUPRAC and ABTS) in comparison with the other methods, and whose chloroform extract was more effective with A_0.50_ = 89.61 ± 0.9 µg/mL for the CUPRAC assay and IC_50_ = 132.66 ± 2.86 µg/mL for the ABTS assay. In all antioxidant assays, the results presented in Table 1 showed that, for the six methods, the moderate activity of the chloroform extract was more effective compared to the other samples. This result may be due to the type and number of volatile compounds present in the samples in addition to the mode of action of each antioxidant method. The antioxidant activity of the plant extracts varies between the assay methods due to the mode of action, the differences in sensitivity of the reagents used in each method, and the complex nature of the phytochemicals present in them, as well as the solvent used for extraction [34]. 

Recent results indicated that oxidative stress can promote AD [3]. Moreover, the inhibition of AChE and BChE constitutes a therapeutic approach for treating the symptoms of AD [5]. Therefore, the purpose of this research was to demonstrate how this plant could inhibit the catalytic site of both AChE and BChE enzymes. The samples demonstrated inhibitory effects in a dose-dependent manner against AChE; the highest anti-AChE activity was found in the essential oil (IC_50_ = 10.66 ± 0.12 µg/mL), which was comparable to the standard galantamine. The nonpolar extract also exhibited inhibitory effects on AChE. The order of activity was essential oil > petroleum ether > hexane > chloroform. High activity against AChE and BChE have been reported for Mentha aquatic aerial parts and roots with chloroform extract (IC_50_ = 20.18 ± 0.55 μg/mL) and acetone extract (IC_50_ = 19.23 ± 1.42 μg/mL) for BChE, and methanol extract (IC_50_ = 20.7 ± 2.11 μg/mL) for AChE [45]. At 200 µg/mL, the acetone and methanol extracts of Mentha longifolia subsp. Noeana possessed a good inhibition against butyrylcholinesterase (58.96 ± 1.72% and 61.69 ± 5.00%, respectively) [45]. The essential oil also exhibited higher activity against BChE (IC_50_ = 16.33 ± 0.03 µg/mL). The inhibitory effect was higher than the used standard, while the petroleum ether, hexane, and chloroform extracts exhibited moderate inhibitory effects. Previous studies suggested that the major components of 1,8-cineole and pulegone identified by the GC-MS of *M. piperita* showed a strong cholinesterase inhibitory effect [44,46]. Moreover, other components identified in *M. piperita* as minor compounds, including α-pinene and camphene terpineol, exhibited interesting AChE-inhibitory abilities [46,47].

Docking verifications were carried out to evaluate the binding affinity and to obtain a better understanding of the binding modes of the major compounds from *M. piperita* against both AChE and BChE. Galantamine was used as an internal reference standard for comparison. Among the seven major compounds from *M. piperita*, N-acetyl-17-oroidine showed the best docking score and thus the best inhibitory potency with both enzyme targets (Table 2). This promising compound was selected for further investigation of its binding mode and amino acid interactions. As shown in Figure 2, N-acetyl-17-oroidine interacted with the catalytic anionic site (CAS) and peripheral anionic site (PAS), unlike to galantamine, which is only bound to the CAS. It should be noted that the best AChE inhibitors bind simultaneously to both CAS and PAS [40,45,48,49,50,51,52,53,54,55,56,57]. N-Acetyl-17-oroidine formed five interactions with the AChE-active site, more than that formed by galantamine (four interactions) (Figure 3). Tyr72 and Tyr337 were found to have two hydrogen bonds along with three π–π stackings that were observed with Tyr124, Tyr337, and Phe286. This last amino acid was said to play a big part in stopping PAS [28]. N-acetyl-17-oroidine is bound to the CAS and PAS of the BChE-active site, thus leading to an inhibitory potency higher than that of galantamine, which is only bound to the CAS (Figure 4). N-acetyl-17-oroidine was involved in three interactions (two π–π stacking with Tyr332 and His438 and hydrogen bond with Asp70), while galantamine was involved in only two places (a π-cation with Trp82 and a hydrogen bond with Glu197) (Figure 5). His438, which formed a π–π stacking with N-acetyl-17-oroidine, is one of the catalytic triad residues of BChE [26]. 

## 5. Conclusions 

This is a preliminary report on the chemical profile and the antioxidant and anticholinesterase capacities of the essential oil and nonpolar extracts of *M. piperita*. A high anti-Alzheimer’s effect was observed in essential oil with IC_50_ values, which is close to galantamine in AChE and more active in BChE. According to the chemical profile analyzed by the GC-MS experiment of the studied samples with their major constituents, the obtained results of the biological activities were supported by modeling experiments that revealed a good affinity of the seven major compounds with the studied enzymes (AChE and BChE), especially N-acetyl-17-oroidine, which showed the best docking score and thus the best inhibitory potency with both enzyme targets, suggesting their potential application as natural inhibitors. Our results showed that the nonpolar extracts and essential oil of *M. piperita* could be used as a source of effective compounds against Alzheimer’s disease.

## Figures and Tables

**Table 1:** Antioxidant activity with phenolic and flavonoid content of nonpolar extracts and essential oil of *Mentha piperita* by DPPH^•^, ABTS^•+^, CUPRAC, reducing power, phenanthroline, and silver nanoparticle (SNP) assays.

Extracts	DPPH^·^ Assay	Reducing Power Assay	Phenanthroline Assay	SNP Assay	ABTS^·+^ Assay	CUPRAC Assay	Total Phenolics (μg GAE/mg) *	Total Flavonoids (μg QE/mg) **
	IC_50_ (µg/mL)	A_0.50_ (µg/mL)	A_0.50_ (µg/mL)	A_0.50_ (µg/mL)	IC_50_ (µg/mL)	A_0.50_ (µg/mL)		
Essential oil	>800	>200	>200	>200	270.91 ± 6.68 ^d^	182.91 ± 1.68 ^h^	22.51 ± 0.14 ^a^	0.66 ± 0.03 ^a^
Petroleum ether extract	>800	>200	>200	>200	>800	167.36 ± 1.38 ^f^	144.81 ± 0.16 ^d^	7.039 ± 0.33 ^b^
Chloroform extract	>800	>200	140.11 ± 0.98 ^e^	124.97 ± 0.41 ^d^	132.66 ± 2.86 ^c^	89.61 ± 0.9 ^e^	109.08 ± 2.85 ^c^	9.29 ± 0.77 ^c^
Hexane extract	>800	>200	>200	>200	>800	170.33 ± 2.89 ^g^	89.09 ± 1.25 ^b^	1.15 ± 0.01 ^a^
BHA ^b^	9.11 ± 0.89 ^c^	5.60 ± 0.05 ^b^	1.49 ± 0.08 ^ab^	73.47 ± 0.88 ^c^	2.98 ± 0.11 ^ab^	2.22 ± 0.15 ^a^	NT	NT
BHT ^b^	1.60 ± 0.36 ^a^	14.48 ± 0.07 ^c^	2.20 ± 0.04 ^b^	>200	1.31 ± 0.06 ^a^	5.53 ± 0.03 ^b^	NT	NT
α-tocopherol ^b^	19.99 ± 0.74 ^d^	>200	5.78 ± 0.30 ^c^	63.41 ± 4.39 ^b^	10.54 ± 0.07 ^b^	17.56 ± 0.17 ^d^	NT	NT
Quercetin ^b^	3.40 ± 0.30 ^b^	4.03 ± 0.38 ^a^	0.65 ± 0.04 ^a^	11.25 ± 0.78 ^a^	2.50 ± 0.06 ^a^	2.42 ± 0.1 ^a^	NT	NT
Ascorbic acid ^b^	2.69 ± 0.22 ^b^	5.60 ± 0.05 ^b^	8.30 ± 0.76 ^d^	>200	4.04 ± 0.02 ^ab^	10.98 ± 0.14 ^c^	NT	NT

The concentration at 50% inhibition and the concentration at 0.50 absorbance, respectively, are referred to IC_50_ and A_0.50_ values. By using a linear regression analysis, the IC_50_ and A_0.50_ values were determined and expressed as mean ± SD (n = 3). The values in the same columns that have different superscripts (a, b, c, d, e, f, g, or h) differ significantly (*p* < 0.05). BHA: butylated hydroxyanisole, BHT: butylated hydroxytoluene, b: reference compounds, BHA: butylated hydroxyanisole, BHT: butylated hydroxytoluene, Nd: not determined, NT: not tested, * total phenolics (μg gallic acid/mg extract), ** total flavonoids (μg quercetin/mg extract).

**Table 2:** Docking scores of galantamine and major compounds from *Mentha piperita* with both AChE and BChE.

Compound	Docking Score (Fitness)	
	AChE	BChE
Imidazoquinoline	45.36	39.23
N-acetyl-17-oroidine	63.01	63.68
1,8-Cineole	37.16	30.81
n-Eicosane	36.30	43.08
l-Menthone	38.76	36.05
Linolenic acid	35.77	43.57
Pulegone	37.45	36.25
Galantamine	57.02	53.04

**Table 3:** Molecular docking scores and interactions of N-acetyl-17-oroidine with AChE- and BChE-active site.

Enzyme	PDB ID	Docking Score	Interactions Formed in the Active Pocket	
			Type of Interactions	Residue Information
AChE	4M0E	63.01	Hydrogen bond π–π stacking	Tyr72, Tyr337 Tyr124, Trp286, Tyr337
BChE	2XQF	63.68	Hydrogen bond π–π stacking	Asp70 Tyr332, His438

## Data Availability

The data are available from the corresponding author.

## Supplementary Materials

The following supporting information can be downloaded at: https://www.mdpi.com/article/10.3390/foods12010190/s1, Table S1: Chemical composition, retention indices, and percentage composition of essential oil of *Mentha piperita*; Table S2: Chemical composition, retention indices, and percentage composition of petroleum ether extract of *Mentha piperita*; Table S3: Chemical composition, retention indices, and percentage composition of the chloroform extract of *Mentha piperita*; Table S4: Chemical composition, retention indices, and percentage composition of hexane extract of *Mentha piperita*.
