# Supplementary material for: Evaluation of In Vitro and In Silico Anti-Alzheimer Potential of Nonpolar Extracts and Essential Oil from Mentha piperita
Source: Foods. 2023 Jan 1;12(1):190. doi: 10.3390/foods12010190 (PMC9818812; doi:10.3390/foods12010190)
Supplement: Supplementary file 1 [file foods-12-00190-s001.zip › foods-1984842-supplementary.pdf]

Table S1. Chemical composition, retention indices, and percentage composition of essential oil of Mentha piperita.

Pic	Compound[a]	TR	RI[b]	(%)
1	Dichotine, 19-hydroxy-11-methoxy-,triacetate (ester)	3.782	4188	0.005
2	2(1H)-Pyridinone, 1,3,4,5,6-pentamethyl-	4.508	1363	0.009
3	Butanamide, N-(5,6,7,9-tetrahydro-1,2,3,10-tetramethyloxy-9-oxobenzo(a)heptalen-7-yl)-, (S)-	5.051	3455	0.008
4	syn-4,7,14,17-Tetrachloro[2.2](5,8)phthalazinophane	5.868	3456	0.009
5	[1,1'-Bianthracene]-9,9',10,10'-tetrone, 2,2',4,4',5,5',8-heptahydroxy-7,7'-dimethyl-	6.148	5689	0.007
6	Triimidazo[1,5-a:1',5'-c:1",5"-e][1,3,5]triazine-1,5,9(2H,6H,10H)-trione, 2,6,10-tris(1,1-dimethylethyl)hexahydro-3,3,7,7,11,11-hexamethyl-	6.474	3545	0.013
7	Dibenzo(a,f)cyclopropa(cd)pentalene, 1,2,3,4-tetrachloro-4b,8b,8c,8d -tetrahydro-	7.308	2852	0.012
8	α-Pinene	8.211	931	0.516
9	Isoxazol, 4-bromo-3-phenyl-5-[(5'-morpholino-3'-phenyl)isoxazol-4'-yl]-	8.417	3444	0.006
10	2-Pentenedinitrile	9.126	1239	0.045
11	N,N'-[6-Methylpyrido[3,2-d]pyrimidine-2,4-diyl]bisbenzamide	10.532	3699	0.086
12	Lanostan-18-oic acid, 3β,11β-dihydroxy-, γ-lactone, acetate	10.652	3240	0.006
13	Tricyclene	10.806	922	0.245
14	5-Azabicyclo[2.2.0]hex-2-en-6-one	11.583	767	0.069
15	2-Methyl-1,4-pentadiene	11.629	580	0,073
16	(E)-2-Heptenal	12.549	942	0.018
17	Oxirane, 2-(chloromethyl)-2-(1,1-dimethylethyl)-	12.629	922	0.079
18	Imidazo(1,5-a)pyrimidine	14.012	1066	0,065
19	Chloromethyl 5-chloropentanoate	14.064	1648	0.019
20	DL-Limonene	14.241	1026	0.549
21	Camphene	14.327	943	0.130
22	1,8-Cineole	14.778	1023	9.643
23	Sarcosine, N-(cyclopentylcarbonyl)-, pentadecyl ester	16.276	2818	0.021
24	Thiourea, N-phenyl-N'-(2-phenylethyl)-	16.304	2449	0.019
25	Benzisoxazole-2-acetic acid, hydrazide	17.522	1888	0.298
26	(3-Oxo-3H-benzo[f]chromen-1-yl)-acetic acid ethyl ester	19.374	2480	0.009
27	Brocresine	19.734	1653	0.103
28	Pyrazophos	19.859	2569.1	0.083

29	Tricyclo[3.2.2.0]nonane-2-carboxylic acid	19.905	1326	0.065
30	Phenyl 5-methoxypentanoate	19.985	1534	0.025
31	Cyclohex-2-enone, 3-(2H-tetrazol-5-ylamino)-	20.048	2329	0.049
32	*p*-Nitrophenylhydrazine	20.151	1531	0.117
33	Myrcene	20.202	979	0.100
34	2-Propanone, 1-phenyl-, oxime	21.157	1325	0.013
35	Carbonic acid, chloromethyl (4-methylphenyl) diester	21.203	1475	0.008
36	Acetic acid, 17-(4-chloro-5-methoxy-1,5-dimethylhexyl)-4,4,10,13,14-pentamethyl-	21.231	3268	0.012
37	p-Toluenesulfonyl-tyrosyl-S-methylcysteine, ethyl ester	21.311	3991	0.045
38	4-Hydroxyadamantan-2-one	21.700	1240	0.024
39	2,8-Decadiyne	21.894	1050	0.031
40	Furfurylideniminosulphur pentafluoride	21.940	966	0.016
41	Nortricyclen	22.014	857	0.019
42	2,4-Hexadien-1-ol	22.460	916	0.017
43	2,4,6-Cycloheptatrien-1-one, 2,3-dimethyl-	22.614	1136	0.050
44	5,6,7-Trinitro-1,4-benzodioxane	23.020	2331	0.180
45	Furazan-3-amine, 4-(1,2,4-triazol-3-yl)-	23.106	1690	0.288
46	*p*-Menthone	23.706	1133	0.218
47	Isomenthon	23.866	1146	43.762
48	4-Hexen-2-one, 3-methyl-	23.986	797	0.078
49	*endo*-Borneol	25.472	1148	0.259
50	1-(1-Butyny)cyclopentanol	25.621	1127	0.561
51	Terpinen-4-ol	25.792	1161	0.340
52	Pyridine, 2,4-dimethyl-	26.695	903.9	0.173
53	Benzeneethanamine	27.461	1136	0.044
54	2,3-Diformylphenol	27.827	1504	0.060
55	2-Methylenebicyclo[2.2.1]-heptane	27.918	808	0.040
56	2-Butene, 1-chloro-4-ethoxy-	27.981	927	0.041
57	Dansyl-L-methionine	28.238	3252	0.158
58	7,8-Dioxabicyclo(4,2,2)dec-9-ene	28.358	1061	0.074
59	2-Pyrazolin-3-amine, N-(3,5-dichloro-2-methoxybenzylidene)-1-phenyl-	28.713	2833	0.013
60	Tetrazolo[1,5-a]pyrimidine, 5,7-dimethyl-	29.250	1636	0.027
61	Pulegone	30.359	1216	21.610

62	*cis*-Piperitone oxide	31.187	1230	3.771
63	Piperitone	31.376	1228	1.083
64	Hexan-1,6-diol, O,O'-bis[4-aminophenyl]-	31.673	2739	0.054
65	(1R,2R,8aS)-2,4,4,7a-Tetramethyl-1-(3-oxobutyl)-trans-hydrindan-2-carboxylic acid	32.188	2176	0.047
66	2-Amino-4,6-bis(3-pyridyl)-1,3,5-triazine	32.336	2335	0.082
67	2,3-Dimethoxybenzamide	32.588	1581	0.062
68	Benzene, 1-methoxy-4-methyl-2-[[[2-(4-nitrophenyl)hydrazino]carbonyl]amino]-	32.942	2962	0.049
69	3-Methylenecyclohexene	33.388	768	0.103
70	3-Methyl-2-nitrophenol	33.657	1410	0.081
71	Carvacrol	34.280	1278	0.424
72	2,4,6-Trimethyl-1,3-phenylenediamine	34.508	1318	0.092
73	1H-Pyrido(4,3-b)indole, 2,3,4,5-tetrahydro-2-cyclohexyl-5-(2-(6-methyl-3-pyridyl)ethyl)-	35.057	3133	0.025
74	2-Isopropylidene-3-methylhexa-3,5-dienal	36.777	1141	1.368
75	Sarcosine, N-(cyclohexylcarbonyl)-, tetradecyl ester	37.692	1845	0.082
76	Benzene, 1-methyl-4-(methylthio)-	38.252	1158	1.568
77	2,4-Cyclopentadiene-1-ethanamine	38.606	967	0.146
78	1-Pentyne, 3-methoxy-3-methyl-	38.823	706	0.221
79	1-Phenyl-2-[4-(2-phenyloxazol-5-yl)phenyl]ethyne	39.109	2820	0.059
80	2,3-Disila-2,2,3,3,6,10-hexamethylundeca-5,9-diene, 4-(phenylthio)-	39.503	2162	0.076
81	Zymosterol	40.195	2674	0.089
82	Ylangene	40.887	1372	0.403
83	*m*-Toluamide	41.487	1316	0.095
84	Alloaromadendrene	42.058	1490	0.369
85	2-Methoxy-5-nitrophenol, heptafluorobutyrate	42.807	1401	0.063
86	2-Bornanone oxime	43.150	1282	0.128
87	5,6,7-Trinitro-1,4-benzodioxane	43.367	2331	0.266
88	Androstan-17-one, 3-methoxy-16,16-dimethyl-, (3β,5α)-	44.047	2212	0.068
89	Germacrene-d	44.785	1480	0.519
90	Benzamide, 4-chloro-N-(2,4-dimethoxyphenyl)-	46.362	2425	0.039
91	5,5-Dimethyl-2-phenyl-1,3-oxazin-4-one	46.756	1698	0.102
92	Benzene, 1,2-diethyl-	47.036	1067	0.086
93	Benzotrichloride	47.254	1187.4	0.080

94	4-Pentenal, 2-methylene-	49.105	763	0.088
95	Naphthalene, decahydro-4a-methyl-1-methylene-7-(1-methylethenyl)-, [4aR-(4aα,7α,8aβ)]-	49.848	1478	0.109
96	4-Normethyl-9,19-cyclolanoststan-7-one, 3-acetoxy-	50.071	3005	0.143
97	Spathulenol	50.946	1569	1.849
98	Ledene	51.334	1520	0.531
99	2-Trimethylstannyl-5-trichloromethyl-6-chlorobicyclo[2.2.1]heptane	51.757	1935	0.319
100	2,4,6(1H,3H,5H)-Pyrimidinetrione, 5-ethyl-5-phenyl-(CAS)	52.272	1849	0.089
101	1-Cyclooctene	52.632	888	0.169
102	2-Methyl-4-hydroxybenzoxazole	53.426	1366	0.099
103	(+)-*epi*-Bicyclosesquiphellandrene	54.569	1435	0.480
104	5-But-2-enyl-4-methyl-2,3-dihydrothiophene 1,1-dioxide	54.964	1561	0.049
105	3,4-Pyridinedicarboxylic acid	55.301	1613	0.459
106	1-Phenyl-5-amino-1,2-pyrazole	55.604	1522	0.102
107	2-Cyclopenten-1-one, 3-methyl-2-(2-pentenyl)-, (Z)-	57.073	1395	0.315
108	Benzaldehyde, 4-diethylamino-, 3,4-dimethylphenoxyacetyl hydrazone	57.359	3044	0.110
109	Cyclotridecanone	65.172	1730	0.282
110	Isoxazole-4-carboxamide, 3-(2-chlorophenyl)-N-(2-methoxybenzyl)-	68.561	2885	0.059
111	7H-1,2,3-Triazolo[4,5-d]pyrimidin-7-one, 2,6-dihydro-2-(4-methoxyphenyl)-	72.213	2138	0.112
112	Trifluoroperazine	79.255	2688	0.046
113	Cyclobutene, 1-cyano-2,3,3-trifluoro-	80.598	641	0.049
	Total compounds (%)	Total		97.821

[a]Compounds listed according to RI
[b]RI (from literature)

Table S2. Chemical composition, retention indices, and percentage composition of petroleum ether extract of Mentha piperita

Pic	Compound[a]	TR	RI[b]	(%)
1	9-(4-Hydroxy-benzylidene)-4-methyl-2H-furo[2,3-h]chromene-2,8(9H)-dione	4.442	3033	0.034
2	4-*n*-Butyl-3-[*n*-octylsulfonyl]nitrobenzene	4.830	2803	0.107
3	1-Phenyleicosane	5.225	2682	0.101
4	2,5,8,11,14,20,23,26,29,32 Decaoxatricyclo [31.3.1.1(15,19)] octatriaconta-1(37),15,17,19(38),33,35-hexaene	5.288	4381	0.055
4	5-(2-Aminoethyl)-1,3-benzodioxole	6.105	1460	0.062
5	Acetic acid, bromo-, methyl ester	6.739	793	0.047

6	1-(3,5-Dimethyl-1-adamantanoyl)semicarbazide	7.059	2208	0.228
7	2,4-Dimethoxyphenol	7.157	1318.6	0.558
8	Cyclopropane, 1,1'-ethenylidenebis-	7.900	788	0.448
9	N,N'-(1,5-Naphthalene)bis(2-isopentyl-1-cyclopropanecarboxamide)	8.448	3648	0.090
10	Acetylcodeine	8.814	2480	0.168
11	2-Methyl-2-butenenitrile	9.157	749	0.094
12	Piperonyl alcohol	9.271	1380	0.128
13	Isomenthon	9.374	1146	0.485
14	l-Menthone	9.569	1148	10.957
15	*cis*-Z-.alpha.-Bisabolene epoxide	9.706	1531	0.324
16	1-[13C]-Phenylethylamine	10.026	1069.9	0.155
17	Methyl undecyl ether	10.369	1318	0.439
18	Pulegone	10.700	1216	4.636
19	3-Hexene, 3-ethyl-2,5-dimethyl-	10.883	872	1.813
20	6-Methyl-2H-pyran-2-one	10.957	937	0.317
21	4-Pyrrolidin-1-yl-1,5-dihydro-imidazol-2-one	11.072	1365	0.131
22	2,4-Dimethoxyaniline	11.455	1370	0.439
23	Diphosphoramide, octamethyl-	11.540	1816.3	0.257
24	Carbamic acid, N-(4-chlorophenyl)-, 4-nitrophenyl ester	11.695	2408	0.224
25	Triethylene glycol monochloride	11.786	1237	0.052
26	Pyrazine, methoxy-, 4-oxide	11.952	1470	0.397
27	3-(3-Fluoro-4-methoxyphenyl)-5-[4- (4-propylcyclohexyl)-phenyl]-[1,2,4]oxadiazole	12.038	3052	0.085
28	2,4-Cycloheptadien-1-one, 2,6,6-trimethyl-	12.478	1222.7	0.640
29	Piperitenone oxide	12.478	1363	1.444
30	Ergost-5-enol	12.649	2632	0.142
31	7-Azatricyclo[4.2.2.0(2,5)]deca-3,7,9-triene, 8-methoxy-, exo-	13.404	1030	0.116
32	(2E)-3-Methyl-2-penten-4-yn-1-ol	13.524	843	0.486
33	2-Azatricyclo[4.3.1.1(4,8)]undecane	13.689	1269	0.095
34	(7S,8R,S)-7-Hydroxymethyl-8-ethoxy-cis-bicyclo[4.3.0]-3-nonene	13.952	1504	0.133
35	Methoxypyrazine	14.421	877	0.357
36	10-Nitro-3,8,13,18-tetraethyl-2,7,12,17-tetramethyl-21H,23H-porphine	14.827	4247	0.193
37	Pyridine-4-carbohydrazide, N2-(2-hydroxy-5-methoxybenzylideno)-	14.918	2586	0.099

38	Oxazol-2-thione[4,5-o]ergost-7,22-dien-3-ol, acetate(ester)	15.038	3497	0.038
39	1H-Cycloprop[e]azulen-7-ol, decahydro-1,1,7-trimethyl-4-methylene-, [1ar-(1aα,4aα,7β,7aβ,7bα)]-	15.227	1569	0.565
40	Tricyclo[5.2.1.0(2,6)]decane	15.290	1078	0.339
41	Campesterol	15.438	3276	0.120
42	Rheadan-8-ol, 2,3,10,11-tetramethoxy-16-methyl-, (6α,8α)-	15.684	3039	0.272
43	Ethanone, 1-(3-cyclohexen-1-yl)-	16.147	858	0.097
44	5α-Androstan-17β-ol, 2α,3α-epoxy-3-methyl-	16.353	2042	0.064
45	Indolo[1,7a,7-ab]benzazepin-1-one, 4,12a-etheno-3,4,6,7,12,12a(1H)-hexahydro-	16.581	2105	0.275
46	1,3-Benzenediamine, N,N'-bis(sulfinyl)-	16.656	1630	0.071
47	Ppropanesulfonic acid, 3-iodo-, phenyl ester	16.879	1989	0.130
48	Ethoxzolamide	17.513	2578	0.131
49	Isomorellin	17.839	4140	0.177
50	1,2,4-Cyclopentanetrione, 3,3-bis(3-methyl-2-butenyl)-5-(3-methyl-1-oxobutyl)-	17.953	3100	0.242
51	Trichothec-9-en-8-one, 4-(acetyloxy)-12,13-epoxy-3,7,15-trihydroxy-, (3α,4β,7β)-	18.285	2485	0.233
52	6-Phenyl-4-(trifluoromethyl)isothiazolo[5,4-b]pyridin-3(2H)-one	18.393	1979	0.236
53	Methyl palmitate	18.799	1908	0.270
54	Palmitinic acid	19.382	1972	3.659
55	26,27-Dinorcholesta-5,22-dien-3-ol, (3β,22E)-	19.668	2470	0.192
56	(4E)-4-Nonen-2-yne	20.159	941	0.139
57	Sarcosine, N-(cyclopentylcarbonyl) -, decyl ester	20.262	2321	0.078
58	Methyl linoleate	20.445	2071	0.476
59	Methyl 9,12,15-octadecatrienoate	20.514	2101	0.606
60	2,3-trimethylenenorbornane	20.719	1078	0.325
61	Linolenic acid	21.228	2122	25.628
62	*n*-Octadecanoic acid	21.297	2174	2.268
63	Furan, 3-methyl-	21.640	602.2	0.282
64	Distearyl thiodipropionate	22.200	4781	0.679
65	Tricyclo[4.3.1.0(2,5)]decane	22.794	1003	0.625
66	Cyclohexene, 4-ethenyl-	22.914	826	0.136
67	17-(1,5-Dimethylhexyl)-2,3-dihydroxy-10,13-dimethyl-1,2,3,7,8,9,10,11,12,13,14,15,16,17-tetradecahydrocyclopenta[a]phenanthren-6-one	23.034	2956	0.388

68	Cholestane, 3-thiocyanato-, (3α,5α)-	23.371	2955	0.251
69	3-(7-Acetoxy-4b-methyl-2-oxotetradecahydrophenanthren-1-yl)-propionic acid, methyl ester	23.531	2582	0.189
70	1-Decyl-4(1H)-pyridinimine	23.611	1651	0.308
71	*n*-Nonadecane	23.834	312.43	0.963
72	Lup-1-en-3-one, (+)-	24.206	2782	0.299
73	1,4-Bis(p-tolylsulfonyl)-2,3,5,6-tetrafluorobenzene	24.297	3193	0.109
74	1,1'-Biphenyl, 2,2',3,3',4,5',6,6'-octachloro-	24.434	2502	0.175
75	N-(2,3-Dihydro-1,4-benzodioxin-2-ylmethyl)-2,2,2trifluoroacetamide	24.932	1639	0.173
76	7-Oxa-15,20,24,27-tetraazatetracyclo[13.9.6.2(8,11).1(2,6)]tritriaconta-2,4,6(33),8,10,12,31-heptaene-14,26-dione, 20-acetyl-5-methoxy-, [s-(Z)]-	25.069	4748	0.289
77	3,6-Di[trifluoromethyl]-9-phenanthrylcarbamic acid ethyl ester	25.155	2355	0.150
78	Tritriacontane (CAS)	25.355	500.99	0.890
79	Pyridine-2,6-dicarboxylic acid, bis-benzothiazol-2-ylamide	25.863	4054	0.599
80	Tricosane, 2-methyl-	26.069	2363	0.668
81	Pyrido[2,3-d]pyrimidine, 4-phenyl-	26.189	1900	0.265
82	13-Methylheptatriacontane	26.498	3733	0.578
83	Cholestane-3,6,7-triol, (3β,5α,6β,7β)-	26.612	2962	0.261
84	*n*-Nonacosane	26.778	475.3	2.212
85	Phosphine, chloro(2,4,6-triisopropylphenyl)(2,4,6-tri-t-butylphenyl)-	27.189	3395	0.603
86	6-Chloro-3-ethyl-2-methyl-4-phenylquinoline	27.384	2418	0.161
87	*n*-Triacontane	27.452	488.4	1.021
88	Pentanal, 5-hydroxy-, (2,4-dinitrophenyl)hydrazone	27.927	2639	1.071
89	Hexatriacontane	28.252	3600	1.990
90	N-(3-Chlorophenyl)maleimide	28.584	1827	0.240
91	(±)-α-Tocopherol acetate	28.767	3308	0.614
92	Methoxyacetic acid, 3-tetradecyl ester	29.144	1890	0.708
93	Acetamide, N-[2-[[2-[2-(2-nitrophenyl)ethenyl]phenyl]azo]phenyl]-	29.487	3439	0.178
94	Triacontane, 1-bromo-	29.784	3299	0.768
95	Androst-5-ene-3,7-dione, 4,4-dimethyl-17-trimethylsilyloxy-	29.910	2503	0.332
96	*n*-Eicosane	30.253	345.2	2.946
97	Pyrimidine-4,6-dione, hexahydro-1-(3-methoxyphenyl)-5-(2-pyrrolylmethylene)-2-thioxo-	30.590	3038	0.155
98	Silanamine, N-[2,6-dimethyl-4-[(trimethylsilyl)oxy]phenyl]-1,1,1-trimethyl-	30.739	1625	0.226

99	8H-Imidazo(2,1-f)purine, 8-methyl-7-phenyl-	30.922	2155	0.740
100	3-Ketours-12-ene	31.116	2856	1.141
101	Norpluvine diacetate	31.465	2527	0.565
102	2-Pentanone, 3-[(acetyloxy)methyl]-3,4-dimethyl-, (.+-.)-	31.567	1170	0.831
103	5-Methyl-2-phenyl-[1,3]dioxane-5-carboxylic acid (furan-2-ylmethyl)-amide	31.790	2488	0.717
104	17-Isoprogesterone	32.270	2247	1.259
105	2-Amino-1,1-dicarboxylic acid, N-[3-fluorophenyl]-	32.973	1982	0.617
106	3-Pyrroline-3-carboxylic acid, 2-(4-bromophenyl)-4-hydroxy-5-oxo-1-phenyl-, tert-butyl ester	33.922	3130	0.618
107	Isobenzofuran-1,3-dione, 4,5-dimethoxy-	34.305	1822	0.253
108	2-Methyl-3-chloro-6,7-diphenylimidazo[1,2-b]-1,2,4-triazine	34.528	2686	0.108
109	3-Amino-2-phenazinol ditms	34.888	2488	0.044
110	Silane, [[(5α,11β)-androsta-2,16-diene-11,17-diyl]bis(oxy)]bis[trimethyl-	35.134	2253	0.192
111	Piperidine, 1-[2-(4-chlorophenyl)-3-(dimethylamino)-1-thioxo-2-propenyl]-	35.625	2503	0.264
112	2-Isopropyl-5-methylphenyl 2,2,3,3,3-pentafluoropropanoate	37.146	1216	0.451
113	2,3,4,5,6-Pentachloroaniline	37.500	1809.5	0.155
114	Heptasiloxane, hexadecamethyl-	37.974	1437	0.448
115	Cyclodecasiloxane, eicosamethyl-	38.986	2067	0.286
116	Bis[4-[3-methoxypropylamino]-3-aminophenyl]sulfone	39.855	3869	0.162
117	Baccharan-3β-ol	40.895	2853	0.602
118	Benzoic acid ethyl ester, 4-(1,1-dioxo-2,3-dihydro-1H-1.lambda.(6)-thiophen-3-ylamino)-	41.455	2683	0.245
	Total compounds (%)	Total		94.367

[a]Compounds listed according to RI
[b]RI (from literature)

Table S3. Chemical composition, retention indices, and percentage composition of the chloroform extract of Mentha piperita

Pic	Compound[a]	TR	RI[b]	(%)
**1**	1,8-Cineole	7.158	1023	0.715
**2**	Isopulegone	9.524	1159	4.487
**3**	3-Nitro-4-(2,2,3,3,4,4,5,5-octafluoro-pentyloxy)-benzoic acid	9.736	1676	0.657
**4**	Pulegone	10.684	1216	2.329
**5**	2-Furanmethanol	10.867	819	1.728

**6**	5-Bromo-7-methylisatin	11.170	1814	1.204
**7**	8H-5,12b-(Iminoethano)-1H-phenanthro[3,2-d][1,3]dioxin, 2,3,4,4a,5,6-hexahydro-15-methyl-, [4aR-(4aα,5α,12bα)]-	12.490	2277	1.582
**8**	3-Methoxy-2-phenylcinchoninamide	14.902	2624	0.935
**9**	3,5-Dodecadiyne, 2-methyl-	15.222	1284	2.218
**10**	Benz[c]oxane-1,3-dione, 4-(2-furfurylideno)-	15.314	2170	0.918
**11**	1-Cyclohexene-1-methanol	19.286	1732	2.551
**12**	Imidazoquinoline	20.995	4792	7.767
**13**	Decyl disulfide	21.441	2511	1.534
**14**	7-Chloro-1-[heptylimino]-1,3,4,10-tetrahydro-10-hydroxy-3-[4-(trifluoromethyl)phenyl]-9(2H)-acridone	22.235	3563	0.759
**15**	Methyl 2-chloro-3-methylbutanoate	24.053	862	1.133
**16**	Acetamide, 2-(adamantan-1-yl)-N-(1-adamantan-1-ylethyl)-	25.127	2545	0.686
**17**	Sarcosine, N-(cyclohexylcarbonyl)-, dodecyl ester	25.339	2640	1.151
**18**	Corynan-17-ol, 18,19-didehydro-10-methoxy-	26.590	2667	1.732
**19**	15,19-Dimethylpentatriacontane	26.756	3571	1.811
**20**	5H-Isoindolo[1,2-b][3]benzazepin-5-one, 7,8,13,13a-tetrahydro-10-hydroxy-3,4,12-trimethoxy-	27.351	2920	3.177
**21**	Fluphenazine enanthate	27.922	3801	3.368
**22**	18-Pentatriacontanone	28.231	3636	3.800
**23**	Ergosterol	28.637	2650	1.840
**24**	5-Methyltricosane	29.140	2343	1.733
**25**	2,3,5,6-Tetrafluorophenyl isothiocyanate	29.311	1143	0.753
**26**	Eicosane	29.757	345.2	1.686
**27**	Tridentochinon acetate	29.888	3529	0.883
**28**	Itaconamide	30.191	1304	2.786
**29**	Aminoglutethimide, N,N,N,O-tetrakis(trimethylsilyl) deriv.	30.837	2692	0.741
**30**	D-Lysergic acid monoethylamide	30.911	2559	1.248
**31**	Betamethasone dipropionate	31.100	3331	2.105
**32**	Phosphonic acid, [[3,5-bis(1,1-dimethylethyl)-4-hydroxyphenyl]methyl]-, diethyl ester	31.774	2181	1.903
**33**	N,N-Dimethyl-N'-(10-propyl-10H-acridin-9-ylidene)-benzene-1,4-diamine	32.083	3053	1.665
**34**	2,3,7,8,12,13,17,18-Octaethyl-21H,23H-porphin	32.249	4273	0.440
**35**	Cholestan-3-one, 2-(1-methylethyl)-, (2α,5α)-	32.717	2765	0.294

**36**	Cholestan-3-one, 2-(dimethylamino)-, (2β,5α)-	33.523	2779	0.305
**37**	Isomenthon	34.169	1146	1.451
**38**	Indolo[2,3-a]naphtho[1,2-c]cyclopenten-3(8H)-one, 1,2,4,4a,5,6,6a,7,12c,12d-decahydro-12c,12d-dimethyl-4-(4-methyl-2-pentenyl)-	34.369	2954	0.996
**39**	Methyl (13E)-6-(methoxyimino)-9,11,15-trihydroxy-prost-13-en-1-oate, 3TMS derivative	35.512	3368	1.484
**40**	1,3,5-Triethyl-1-(ethylbutoxysiloxy)cyclotrisiloxane	35.815	1585	0.611
**41**	2-(4-Chlorophenyl)-5,7-dimethylimidazo[1,2-a]pyridine-8-carbonitrile	39.302	2385	0.844
	Total compounds (%)	Total		70.01

[a]Compounds listed according to RI
[b]RI (from literature)

Table S4. Chemical composition, retention indices, and percentage composition of hexane extract of Mentha piperita

Pic	Compound[a]	TR	RI[b]	(%)
1	Fluphenazine	3.717	3045	0.087
2	6-Chloroquinoxalino[2,3-b]cholestane	4.911	3563	0.159
4	Pyrimidine, 2,4-dimethyl-	7.163	894	0.485
5	Silane, diethylbis(pentafluorophenyl)-	7.906	1424	0.371
6	l-Menthone	9.375	1148	6.976
7	Phenol, 4-nitro-	10.369	1527.2	0.329
8	Pulegone	10.690	1216	3.620
9	Methylethylmaleimide	10.872	1280.6	1.382
10	3-Hydroxyiminocyclopentan-2-one-1-carboxylic acid	10.970	1521	0.315
11	*p*-Cymene, 2-chloro-	11.461	1221	0.840
12	5-Diazouracil	12.478	1448	1.112
13	[1,2,4]Triazolo[4,3-a]quinoxaline, 1,4-dimethyl-	13.536	1748	0.480
14	Cyclohexene,1-propyl-	14.445	990	0.592
15	5H-[1,2,4]Triazolo[3,4-b][1,3]thiazin-5-one, 7-(4-methoxyphenyl)-	15.228	2285	0.757
16	2,2,4-Tri(4-morpholinyl)-6-(trichloromethyl)-1,3,5,2.lambda.<5>-triazaphosphinine	16.445	3384	0.390
17	30-Norlupan-28-oic acid, 3-hydroxy-21-methoxy-20-oxo-, methyl ester, (3β)-	17.834	3321	0.476
18	Stigmast-7-en-3-ol, acetate, (3β,5α)-	17.971	2871	0.212
19	5,6-Dimethoxyphthalaldehydic acid	18.508	1830	0.286
20	Palmitinic acid	19.308	1942	2.623

21	2-Acetylpyridine 4,4-dibenzylthiosemicarmazone	19.497	3358	0.230
22	Genistein	20.446	2634	0.468
23	*n*-Propyl linolenate	20.509	2300	0.499
24	Linolenic acid	20.714	2134	19.607
25	Ethanone, 1-[4-(chloromethyl)-2-(4-methylphenyl)-5-thiazolyl]-	21.406	2159	0.328
26	4',6-Dichlorothioflavone	22.235	2484	0.350
27	Epiandrosterone	22.469	2128	0.627
28	Ethanol, 2,2,2-trichloro-	22.806	850	0.749
29	2,3,3',4',5,6-Hexachloro-1,1'-biphenyl	22.995	2322.5	0.668
30	Cholest-5-ene, 3-ethoxy-, (3β)-	23.246	2645	0.976
31	Prostaglandin F1α-trimethylsilyl methyl ester	23.458	3000	0.361
32	Pirenzepine, 8-sulfamoyl-	23.458	3827	0.328
33	Octanoic acid, 6-hydroxy-8-methoxy-, ε-lactone	23.829	1401	0.582
34	7H-Furo[3,2-g][1]benzopyran-7-one, 9-[2,3-bis(acetyloxy)-4-(2,5-dihydro-4-methyl-5-oxo-2-furanyl)-3-methylbutoxy]-	24.195	3719	0.547
35	*n*-Butoxycarbonyldemecolcine	24.390	3619	0.564
36	2-Docosenoic acid, 2,4,21,21-tetramethyl-, methyl ester, (E)-	24.595	2709	0.567
37	Chol-8(14)-en-24-ol	24.858	2445	0.509
38	Ethyl 6-bromo-7-hydroxy-2-ureidobenzo[b]thiophene-3-carboxylate	24.932	2863	0.482
39	Nandrolone decanoate	25.064	3000	0.287
40	*n*-Tricosane	25.344	388.7	0.851
41	Chenodiol	25.613	2896	0.416
42	7-Hydroxytomatidine, O,O,N-triacetate	26.058	3660	0.562
43	Cyclopropanecarbonitrile, 1,2-bis(p-nitrophenyl)-	26.493	2739	0.434
44	*n*-tetracosane	26.761	402.5	2.274
45	Estra-1,3,5(10)-trien-15-one, 3,17-bis(acetyloxy)-, 15-(O-methyloxime), (17β)-	27.253	2837	0.460
46	Heneicosane, 3-methyl-	27.447	2182	0.966
47	5,8-Quinolinedione, 7-(heptadecylthio)-6-hydroxy-	27.922	3545	0.746
48	Benzaldehyde, 3,4-methylenedioxy-2-nitro-, thiosemicarbazone	28.019	2676	0.290
49	4,8-Dimethylheptacosne	28.236	2776	2.260
50	Cyclodecasiloxane, eicosamethyl-	28.476	2067	0.184
51	Benzoic acid, 2,5-dichloro-3-hydroxy-6-methoxy-	28.539	1920	0.608
52	Cyclopentanecarboxamide, 3-ethenyl-2-(3-pentenylidene)-N-phenyl-,[1α,2Z(E),3α]-	28.768	2375	0.592

53	Equilin	28.870	2163	0.706
54	4-[2-(2,5-Dioxo-pyrrolidin-1-yloxycarbonyl)-cyclohexanecarbonyl]-piperazine-1-carboxylic acid tert-butyl ester	29.128	3421	1.457
55	Zeranol, tris(trimethylsilyl) ether	29.962	3254	0.498
56	*n*-Eicosane	30.214	345.2	3.701
57	Androst-5-en-7-one, 3,17-bis(acetyloxy)-19-(methoxymethoxy)-4,4-dimethyl-, (3β,17β)-	30.516	3092	0.375
58	Thiocolchicine, 3,N-diethoxycarbonyl-3-demethyl-N-deacetyl-	30.625	3862	0.495
59	Isonipecotic acid, N-(2,4,5-trifluoro-3-methoxybenzoyl)-, dodecyl ester	30.762	3356	0.231
60	Butyric acid, 4-(4-chloro-5-methyl -3-nitropyrazol-1-yl)-	31.088	1997	2.499
61	1h-Pyrrole-3,4-diacetic acid, 2-acetoxymethyl-5-methoxycarbonyl-, dimethyl ester	31.214	2433	0.165
62	4-Nitro-4'-chlorodiphenylsulfoxide	31.488	2347	2.607
63	1,1,1,5,7,7,7-Heptamethyl-3,3,5-tris (trimethylsiloxy) tetrasiloxane	31.768	1437	1.476
64	5-Amino-2-(4-chlorophenyl)-7-methyl-6-indolizinecarbonitrile	31.962	2491	0.443
65	1,4-Dioxa-7,18-diazacycloeicosane-6,19-dione, 7,18-dihexyl-	32.214	4011	1.136
66	Octadecanoic acid, octyl ester	32.334	2773	1.316
67	Cyano[(1H-tetrazol-5-yl)hydrazono]acetic acid, ethyl ester	32.740	2286	1.391
68	Dodecanoic acid, 2,2,3,3,4,4,5,5,6 ,6,7,7-dodecafluoroheptyl ester	32.968	1265	1.568
69	D-Gluconic acid, δ-lactone	33.146	1813	0.380
70	β-Tocopherol, TMS derivative	33.243	2987	0.799
71	1,1'-Biphenyl, 2,2',3,3',5,5',6,6'-octachloro-	33.574	2381	0.588
72	Griseoviridin	33.666	4195	0.178
73	Cholestan-3-one, oxime, (5α)-	33.740	2730	0.281
74	Acetic acid, 17-(4-chloro-5-methoxy-1,5-dimethylhexyl)-4,4,10,13,14-pentamethyl-2,3,4,5,6,7 ,10,11,12 ,13,14, 15,16,17 -tetradecahydro-1-phenanthryl-	33.860	3268	0.672
75	6-Chloro-12H-tetrachlorodibenzo[d,g][1,3,2]phosphorin-6-sulfide	34.014	2883	0.313
76	1,2-Difluoro-1,2,3,3,4,4,5,5,6,6-decamethylhexasilinane	34.249	334	0.639
77	2,3,4-Trihydroxybenzoic acid, 4TMS	34.397	1932	0.644
78	4(1H)-Pteridinone, 5,6,7,8-tetrahydro-6-methyl-	34.597	1830	0.443
79	Spiro[9,9']difluorene, 2,2'-(2,5,8,11-tetraoxadodecane-1,12-diyl)-	34.694	4060	0.279
80	2,5-Dibromonitrobenzene	34.895	1715	0.834
81	4''-Dehydroxy-2'',3',3'',4',5,6'',7-hepta-O-methylisoorientin	35.083	3977	0.414
82	Phosphine, 1,2-ethenediylbis[diphenyl-, (E)-	35.335	3173	0.399

83	Furfurylideniminosulphur pentafluoride	35.752	966	0.652
84	allantoin	36.981	1595	1.302
85	Silane,diphenyl-bis -[2-(1,3,5,5,7,7-heptamethyl-2,4,6,8-tetraoxy-1,3,5,7-tetrasilacyclooctyl)ethyl]	37.232	3087	0.190
	Total compounds (%)	Total		89.935

[a]Compounds listed according to RI
[b]RI (from literature)
